# Exploring the Cardiovascular Benefits of Extra Virgin Olive Oil: Insights into Mechanisms and Therapeutic Potential

**DOI:** 10.3390/biom15020284

**Published:** 2025-02-14

**Authors:** Esposito Milena, Mandalà Maurizio

**Affiliations:** 1 Department of Biology, Ecology & Earth Sciences, University of Calabria, 87036 Rende, Italy; milenaesposito17@gmail.com; 2 Department of Obstetrics, Gynecology and Reproductive Sciences, Larner College of Medicine, University of Vermont, Burlington, VT 05401, USA

**Keywords:** extra virgin olive oil, polyphenols, cardiovascular diseases, oleic acid

## Abstract

Cardiovascular diseases (CVDs) are the leading cause of mortality worldwide, driven by complex interactions among genetic, environmental, and lifestyle factors, with diet playing a pivotal role. Extra Virgin Olive Oil (EVOO), a cornerstone of the Mediterranean diet (MedDiet), is a plant-based fat that has garnered attention for its robust cardiovascular benefits, which are attributed to its unique composition of monounsaturated fatty acids (MUFAs), particularly oleic acid (OA); and bioactive polyphenols, such as Hydroxytyrosol (HT) and oleocanthal. These compounds collectively exert antioxidant, anti-inflammatory, vasodilatory, and lipid-modulating effects. Numerous clinical and preclinical studies have demonstrated that EVOO’s properties reduce major modifiable cardiovascular risk factors, including hypertension, dyslipidemia, obesity, and type 2 diabetes. EVOO also promotes endothelial function by increasing nitric oxide (NO) bioavailability, thus favoring vasodilation, lowering blood pressure (BP), and supporting vascular integrity. Furthermore, it modulates biomarkers of cardiovascular health, such as C-reactive protein, low-density lipoprotein (LDL) cholesterol, and NT-proBNP, aligning with improved hemostatic balance and reduced arterial vulnerability. Emerging evidence highlights its interaction with gut microbiota, further augmenting its cardioprotective effects. This review synthesizes current evidence, elucidating EVOO’s multifaceted mechanisms of action and therapeutic potential. Future directions emphasize the need for advanced extraction techniques, nutraceutical formulations, and personalized dietary recommendations to maximize its health benefits. EVOO represents a valuable addition to dietary strategies aimed at reducing the global burden of cardiovascular diseases.

## 1. Introduction

CVDs encompass a group of disorders related to atherosclerosis, affecting heart and blood vessels. CVDs develop in people of all ages, sexes, ethnicities, and socioeconomic levels, representing the leading cause of both mortality and morbidity worldwide [1]. The burden of CVDs on public health is showing an alarming trend, as demonstrated by the approximate 50% increase in the number of deaths registered over the period 1990–2019 (12.1–18.6 million) [1]. The etiology of CVDs is complex and multifactorial, including both non-modifiable and modifiable risk factors, such as hypertension, dyslipidemia, diabetes, and obesity [2]. An important concern in CVDs’ treatment is the insufficient adherence to the treatment plans by patients themselves. This lack of adherence has been observed in both primary [3] and secondary prevention for CVDs, as demonstrated by the PURE study [4]. Interestingly, 40% of CVD cases may be attributed to unhealthy nutritional lifestyles [5,6]; this evidence, in combination with the lack of adherence to prescribed medications, is a clear indicator that nutritional interventions may represent a valuable ally against CVDs.

The MedDiet is considered a healthy diet mainly due to EVOO, the consumption of which has been associated with a 26–46% reduction in the risk of recurrence of CVDs [7,8,9,10,11] and decreased mortality rate [12]. The beneficial effects following EVOO consumption have been related to its antioxidant, immunomodulatory, and inflammatory response regulating mechanisms, as well as regulation of lipoprotein profile and functionality. EVOO represents the main source of fat in the MedDiet, replacing saturated fats, like butter and margarine, frequently used in the Western diet [13]. EVOO is obtained by the physical process of cold-pressing olive fruit, without any other treatment other than washing, decantation, centrifugation, and filtration. The classification of olive oil as ‘extra virgin’ is strictly regulated by the European commission (Commission Regulation (EEC) No. 2568/91 of 11 July 1991 on the characteristics of olive oil and olive-residue oil and on the relevant methods of analysis) and the Trade Standard of the International Olive Council (IOC). To be defined as EVOO, free acidity content must be ≤0.8% (as free oleic acid), peroxide value ≤ 20 mEq O2/kg, and oxidation levels must be minimal (K270 ≤ 0.22; K232 ≤ 2.50; ∆K ≤ 0.01); there must be with a favorable taste (0 defects and a fruity flavor score > 0), and fatty acid ethyl esters (FAEEs) ≤ 30 mg/kg must be present. Further, purity criteria must be met to maintain this certification, as detailed and discussed in the following review [14].

The aim of this narrative review is to synthesize the cardiovascular benefits of EVOO by discussing studies conducted in both the Mediterranean and non-Mediterranean area to outline the global relevance of EVOO in cardiovascular health. In addition, this review will discuss the mechanisms by which EVOO improves arterial, myocardial, and blood health, as well as its role in regulating BP, low- and high-density lipoprotein cholesterol, endothelial function, and glucose levels. Lastly, it will focus on the possibility of using EVOO in clinical practice to mitigate the global burden of CVDs.

**Figure 1:**
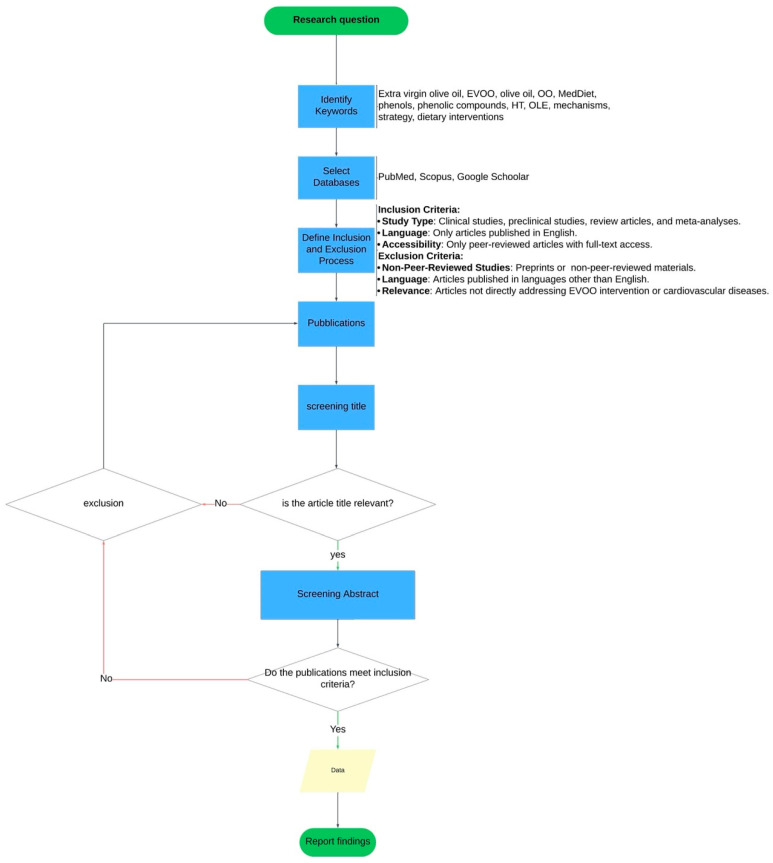
Flowchart of the methodological approach followed for the selection/exclusion criteria of the articles.

This narrative review was conducted following a structured approach, as outlined in Figure 1. Initially, keywords related to the research question were identified, and relevant databases, including PubMed, Scopus, and Google Scholar, were searched. Inclusion criteria focused on clinical studies, preclinical research, review articles, and meta-analyses published in English. Non-peer-reviewed sources and articles not directly addressing the specific research question were excluded. After screening titles and abstracts, eligible publications underwent full-text evaluation to ensure alignment with inclusion criteria. This systematic process resulted in the selection of studies that form the basis of the findings presented below.

## 2. MedDiet Benefits on Cardiovascular Problems

Due to its nutritional completeness, long-term sustainability, and evidence-based health effects, the MedDiet is ranked best diet by US News and World Report (https://health.usnews.com/best-diet/mediterranean-diet, accessed on 21 December 2024). Its health effectiveness is sustained by evidence showing that even a two-point increase in adherence to MedDiet reduces CVDs similar to statin [15]. Several epidemiological studies and clinical trials have demonstrated the association between MedDiet and reduction of CVDs.

The seven-country study launched by Key was the first large-scale study, covering sixteen different communities within seven different countries, over a period of 10 years, showing an association between MedDiet and reduction in cardiovascular risk. They observed a gradient in cardiovascular risk across countries, with Northen European countries experiencing a higher rate of coronary heart disease (CHD) mortality compared to Southern European countries [16]. Since then, a large body of research has proven the association between MedDiet and reduced CVDs risk. For example, Trichopoulou found that people in Greece who more closely followed a traditional MedDiet exhibited a reduction of 25–30% in deaths from CHD and cancer [12]. While the non-Mediterranean population that adheres to the Mediterranean lifestyle experienced only a 10% reduction for both mortality and CVDs [17]. The disparity could be attributed to cultural habits, genetic predispositions, adherence, and other contextual factors.

In addition to observational studies, clinical trials, both in primary and secondary prevention, have established a causal relationship between MedDiet and CVDs. The Lyon Diet Heart study (1988–1992), a clinical trial conducted in France, investigated the adoption of the MedDiet after a first myocardial infarction. In this study, the participants were randomly assigned to follow either the American Heart Association Step One diet which recommended approximately 30% fats, or the MedDiet. Adoption of the MedDiet significantly reduced total mortality by 56%, cardiac mortality by 65%, non-fatal myocardial infarction by 70%, and cancer incidence by 60% [18]. Similarly, Logeril et al., in 1994, found that patients with a previous history of cardiac events experienced a better outcome in both death and new recurrence of cardiac disease following a MedDiet vs. the usual post-infarct prudent diet [19]. In accordance, the secondary prevention trial conducted by Renaud et al. in 1995 demonstrated a 70% reduction in cardiovascular events and total death in people who followed a MedDiet compared to those who followed the usual prescribed diet. These beneficial effects have been related to an increase in n-3 fatty acids and oleic acid, a decrease in linoleic acid, and higher plasma concentrations of antioxidant vitamins C and E [20]. To further validate the findings across these clinical studies, a meta-analysis was conducted by Sofi et al. in 2008 analyzing eight cohorts and involving 514,816 subjects. Adherence to MedDiet was associated with lower cardiovascular mortality (pooled relative risk 0.91), decreased incidence and mortality from cancer (0.94), and neurodegenerative diseases [21]. Further, in a prospective study conducted in the US that involved 25,994 healthy women, the adherence to MedDiet (score of 6 or greater), was associated with a 25% reduction in the incidence of cardiovascular events (stroke, CVD death, and incidence CHD) [22]. Decreased inflammation, insulin resistance, and body mass index have been the primary factors explaining the 25–30% of MedDiet benefits. Additionally, improvements in BP and blood lipids, particularly HDL and very low-density lipoprotein (VLDL), were also involved, as well as LDL particles, in terms of size and particle numbers.

The key studies discussed in this section are summarized in Supplementary Materials Table S1.

## 3. EVOO and Cardiovascular Health: Evidence and Benefits

EVOO, a cornerstone of the MedDiet, has been widely studied for its role in reducing cardiovascular risk. Its consumption has been associated with significant health benefits, as highlighted by several key studies. The recent Harvard study (2021), a prospective cohort study involving 99,000 participants (63,967 women and 35,312 men) over a period of 24 years, found that daily consumption of at least half a tablespoon of EVOO reduced CVDs risk by 15% and CHD by 21%. Further, replacing butter, mayo, dairy, and margarine with EVOO lowered CVDs risk by 5–7% [23]. The PREDIMED (Prevención con Dieta Mediterránea) trial in Spain, involving over 7000 participants high-risk participants, compared three dietary interventions: MedDiet supplemented with EVOO (4 tablespoon/day), MedDiet supplemented with nuts (30 g/day), and a low-fat control diet. Both the EVOO and nuts-supplemented group demonstrated a 30% reduction in major cardiovascular events. The EVOO group also showed additional benefits, including reduced risk of atrial fibrillation, diabetes, breast cancer, and neurocognitive decline [15]. The EPIC-Europe study, a long-term study of over 500,000 participants, across 10 European countries, confirmed the cardiovascular benefits of MUFAs in EVOO, highlighting its protective effects on heart health.

EVOO’s unique composition, high MUFA and polyphenolic compounds, underpins its cardiovascular benefits by improving glucose metabolism, BP, lipid profiles, and obesity, all factors that have been demonstrated to have a strong correlation with CVDs incidence [23,24].

EVOO positively influences glucose metabolism. Type 2 diabetes, characterized by insulin resistance (IR) and hyperglycemia [25,26,27], is a major contributor to cardiovascular events. Studies have consistently demonstrated that EVOO decreased the risk of type 2 diabetes [28,29] by improving postprandial glucose regulation and insulin sensitivity [30] and increasing glucagon-like peptide-1 incretin pattern [31]. Remarkably, phenolic compounds in EVOO have been shown to lower the lipoprotein insulin resistance index (LP-IR) [32,33,34,35,36,37,38], to reduce hepatic release of glucose, and to increase its peripheral uptake [39] and reduce the production of HbA1C [40].

EVOO positively influences BP. Hypertension, a leading modifiable risk factor for CVD [41], is significantly impacted by EVOO. Long-term consumption of EVOO lowers both systolic and diastolic BP, as demonstrated in several studies. For instance, a six-month MUFA-rich diet reduced resting BP and the need for antihypertensive drugs [42]. Further, a study using olive leaf extract also showed substantial antihypertensive effects comparable to conventional anti-hypertensive medications like captopril [43]. Moreover, studies on hypertensive patients demonstrated that phenolic-rich EVOO reduced both systolic and diastolic BP in men [44] and in women [45]. Additionally, replacing corn, soybean oil and butter with EVOO decreased systolic BP in overweight and obese adults [46]. The reduction in BP following EVOO consumption is extremely significant, as a reduction of 2 mmHg in BP correlates with decreased risk of CHD (6%), stroke (10%) and heart attack (15%) [47].

EVOO positively influences obesity. Although studies on EVOO’s impact on weight are mixed, evidence suggests its role in mitigating obesity-related cardiovascular risks. For example, the PREDIMED study found a modest reduction in waist circumference among EVOO consumers over five years [48]. Further, the MedDiet, which is rich in EVOO, has been associated with the prevention of weight gain [49].

EVOO positively influences lipidic profile and reduces atherogenic risk [50,51]. The EUROLIVE study represents a seminal investigation in the context of EVOO effects on lipidic profile. This randomized control trial was conducted on healthy subjects among several European populations. The intervention consisted in the ingestion of 25 mL/daily of olive oils enriched with varying levels of polyphenols. The results of the study demonstrated that phenolic compounds prevent lipoprotein oxidation, and this effect was correlated with the dose [52]. Subsequently, Cicero et al. found that the reduction in the oxidative damages to lipids following EVOO consumption was associated with an increase in MUFA relative to PUFA in the fatty acid composition of the LDL [53]. Further, phenol-rich olive oil enhances HDL function, thus improving cholesterol efflux capacity, increasing HDL size and fluidity, and reducing oxidative stress [54]. Several trials demonstrated reductions in LDL cholesterol and triglycerides, along with shifts in HDL composition toward a less atherogenic profile [44,46].

The key studies discussed in this section are summarized in Supplementary Materials Table S2.

### 3.1. EVOO Impacts on Arterial Health

Arteries are essential conduits of the cardiovascular system, carrying blood from the heart to other districts of the body. Their susceptibility to damage and disease, known as arterial vulnerability, affects blood flow and overall cardiovascular health. Endothelial dysfunction and atherosclerosis are key mechanisms contributing to arterial impairment, substantially increasing cardiovascular risk [55].

EVOO’s role ability in mitigating arterial vulnerability has been demonstrated by several studies and is associated with its positive effects on endothelial function [56]. An early study by Cutruzzolà et al. (2021) demonstrated that an EVOO-enriched meal significantly improved Flow-Mediated Dilation (FMD) compared to a butter-enriched meal in 10 individuals with Type 1 diabetes and 6 healthy controls [57]. Also, a meta-analysis including over 30 studies with 3106 participants found that EVOO consumption increases FMD values, confirming EVOO beneficial effects on endothelial function [58]. The CORDIOPREV study further demonstrated a 2.63% improvement in FMD among CHD patients following a MedDiet rich in EVOO compared to low-fat diet. This improvement was associated with higher endothelial progenitor cells, lower oxidative stress and higher angiogenesis [59]. Additionally, the long-term consumption of a MedDiet rich in EVOO may preserve kidney function, as shown by a reduced decline in eGFR in CHD patients with Type 2 diabetes mellitus (T2DM) [60]. In contrast, research by Vogel (2000) observed a 31% reduction in FMD three hours post-meal containing OO [61]. However, this study was limited to the immediate impact rather than long-term effects. Further, the observed reduction could be influenced by the digestive process during which the increase in the blood-flow digestive apparatus could influence the endothelial function, leading to a temporary reduction of FMD [62].

EVOO consumption also prevents atherosclerotic plaque, as shown by a clinical trial that associated an EVOO-rich MedDiet with a reduction in carotid plaque and carotid intima-media thickness (IMT) compared to a low-fat diet [63]. These findings emphasize the protective role of EVOO in slowing the progression of atherosclerosis. In accordance, a study including 199 subjects showed a significant inverse association between daily OO intake (above 34 g) and IMT [64]. Interestingly, in the PREDIMED study, EVOO consumption did not reduce IMT or plaque size; however, it prevented further plaque progression [65], indicating a stabilizing effect.

The key studies discussed in this section are summarized in Supplementary Materials Table S3.

### 3.2. EVOO Impact on Hemostasis

Alterations in hemostasis including platelet activations [66], coagulability, and fibrinolysis have been frequently associated with CVD risk. A preventive effect on this process may be achieved using EVOO, as demonstrated by several studies. Sirtori et al. (1986) demonstrated that the assumption of 45 g EVOO daily reduced platelets sensitivity to collagen-induced aggregation [67]. Another pilot study found that 21 g EVOO daily for 8 weeks inhibited platelet aggregation and reduced thromboxane A2 (TXA_2_) levels. These effects have been associated with an increased content of OA in platelet membrane associated with a negative correlation with arachidonic acid content [68]. This finding is supported by evidence that a MUFA-rich diet diminished urinary excretion of thromboxane B2 (TXB_2_) metabolite [69], indicating diminished platelet activation. In addition, more recent findings have demonstrated the ability of OA to lower the aggregation to arachidonic acid, collagen and ADP [70]. Interestingly, also phenolic compounds have been associated with lower plasma levels of TBX2 [71].

However, the effects of EVOO’s on coagulation factors such as Factor VII (FVII) and von Willebrand factor (vWF), a marker of platelet adhesion and coagulation pathways, have shown inconsistent results. For instance, EVOO has been shown to decrease vWF, which favors the adherence of platelets to the sites of vascular injury [72]. Conversely, studies investigating the effect of EVOO on FVII have yielded mixed findings: one study reported that MUFAs could increase activated FVII levels [73], while another found no significant effect on FVII [74]. The discrepancies could be attributed to differences in experimental designs, dietary comparisons, and intervention duration. Specifically, one study examined chronic effects while the other focused on acute responses. Moreover, one study compared diets rich in OA (MUFA) and linoleic acid (PUFA), both unsaturated fatty acids, possibly explaining the similar long-term effects on coagulation pathway. In contrast, the other study introduced a saturated fat component, which is known to elicit a stronger postprandial lipidemic and inflammatory response, potentially leading to more pronounced changes in coagulation markers. Thus, further studies are required to elucidate the effects of EVOO consumption on hemostatic cascade.

Fibrinolysis, the process preventing thrombus formation, is regulated by tissue plasminogen activator (tPA) and its inhibitor, PAI-1. Contrasting evidence exists regarding EVOO’s effect on PAI-1 levels, with reductions observed in some studies [75,76] but not in others [77], possibly due to variations in participants’ metabolic status or control diets. Interestingly, oleuropein, a phenolic compound in EVOO, acts as a natural inhibitor of PAI-1 in human breast cancer cells [78].

In summary, most of the research supports the role of EVOO in reducing the major modifiable cardiovascular risk factors. Although some controversial results have been reported, they may stem from differences in study design, sample size, dietary intervention, EVOO composition, genetic background, and lifestyle habits of the participants.

The key studies discussed in this section are summarized in Supplementary Materials Table S4.

## 4. Cardiovascular Biomarkers and EVOO’s Effects

Cardiovascular biomarkers are crucial in the diagnosis and management of CVDs. Cardiovascular biomarkers are released into the blood stream following damage or a stress in the cardiovascular system. They are measurable indicators of physiological or pathological processes and are essential for assessing CVD risk and progression [79]. This section provides a comprehensive overview of cardiovascular biomarkers, categorized by their role in CVD risk assessment, along with the effects of EVOO on these biomarkers.

### 4.1. Cardiac Injury Markers

*Aspartate/alanine aminotransferase (AST/ALT)*: AST and ALT are primary liver enzymes; however, they also reflect cardiovascular health [80,81,82]. An elevated AST/ALT ratio has been associated with heart failure [83], cardiovascular mortality in hypertensive patients [84], insulin resistance [85], and arteriosclerosis [86]. Reduced levels of AST and ALT following EVOO consumption have been demonstrated in both animal [87] and human trials [88]. This effect has been mainly attributed to MUFA [89]. However, some findings remain inconsistent [90].

*Lactate dehydrogenase (LDH)*: LDH is a marker of oxidative stress, inflammation, and endothelial dysfunction [91,92]. Increased levels of LDH have been associated with myocardial infarction. Animal studies have demonstrated that VOO consumption reduces LDH levels [87,93], highlighting its potential cardioprotective effects.

*Creatine kinase-MB (CK-MB)*: CK-MB is a specific marker for myocardial damage, and its levels increase post-myocardial infarction [94]. Further, its elevation correlates with a greater atherosclerotic plaque burden after coronary interventions [95]. EVOO has been shown to reduce CK-MB levels in animal models of cardiotoxicity [93], indicating a protective role against myocardial injury.

*Cardiac troponins* (cTNT, cTNI, and cTNC): Elevated levels of troponins are critical markers of cardiovascular risk and myocardial injury [96,97]. High concentrations of cTN have also been correlated with artery calcification [98], altered lipid metabolism [99], and endothelial dysfunction [100]. An animal study showed that EVOO administration lowered troponins levels [101], supporting its potential to mitigate cardiac damage.

*N-terminal pro B-type natriuretic peptide (NT-proBNP)*: NT-proBNP levels have been strongly associated with increased risk of cardiovascular disease [102,103,104,105,106]. A clinical trial demonstrated that a MedDiet rich in EVOO decreased NT-proBNP level under the 80th percentile, considered a threshold for CVD mortality risk [107], suggesting its role in alleviating cardiac stress.

### 4.2. Inflammatory Markers

*C-reactive protein (CRP) and interleukin-6 (IL-6)*: CRP is a key inflammatory marker associated with cardiovascular risk [108]. Once released into circulation, it induces inflammation, disrupting vascular endothelial homeostasis and worsening atherosclerotic events [109]. Besides its role in CRP synthesis, IL-6 has been found to predict cardiovascular events [110], and its levels correlate with an increased risk of CHD [111]. EVOO has been shown to reduce both CRP and IL-6 levels [58], underscoring its anti-inflammatory properties.

*Tumor necrosis factor*-α *(TNF-α):* TNF-α contributes to inflammation, endothelial dysfunction, atherosclerosis, and insulin resistance [112,113]. Several studies in animals reported that EVOO reduced TNF-α [114,115]. Also, the PREDIMED study showed that a MedDiet rich in EVOO reduced TNF-α levels [7]; however, results from meta-analysis remain inconclusive [58].

*Soluble CD40 ligand* (sCD40L): sCD40L, a member of the TNF-α family, is linked to platelet activation, atherosclerosis, and endothelial dysfunction [116,117,118]. Its role in CVD development is further underscored by its elevated levels in coronary artery disease [119]. sCD40L is reduced by the two major EVOO polyphenols, HT and oleuropein (OLE) [120], highlighting its anti-atherogenic potential.

### 4.3. Fibrosis and Remodeling Markers

*Matrix metalloproteinases (MMPs)*: MMPs are a family of proteolytic enzymes involved in the remodeling of the extracellular matrix. MMPs, specifically MMP-9, are associated with cardiovascular mortality [121], atherosclerosis [122], and insulin resistance [123]. EVOO polyphenols, HT and OLE, have been demonstrated to inhibit MMP-9 activity, thus reducing inflammatory angiogenesis [124].

*MMP9/NGAL complex*: NGAL belongs to the lipocalin family and is a marker of kidney health. The MMP9/NGAL complex contributes to myocardial injury [125], plaque instability [126,127], and collagen degradation [128]. EVOO polyphenols, such as oleacein, inhibit NGAL/MMP9 activity [129], promoting vascular stability.

*Galectin-3 (Gal-3)*: Gal-3, a carbohydrate-binding protein, is associated with elevated risk of cardiovascular mortality and heart failure [130,131]. EVOO has been shown to modulate Gal-3 activity [132], reducing its pro-CVD effects.

### 4.4. Metabolic and Hemostatic Markers

*Trimethylamine N-oxide (TMAO)*: TMAO is a gut microbiota-derived metabolite promoting inflammation [133], atherosclerosis [133,134], endothelial dysfunction [135], lipid homeostasis [136], and insulin resistance [137], all factors which trigger increased CVD risk [138]. A study in animals demonstrated that EVOO inhibits TMAO formation [139]; however, data on humans are controversial [140,141,142,143].

*Adiponectin*: Adiponectin is secreted by adipocyte. It reduces inflammation and oxidative stress and promotes endothelial function. Low levels of adiponectin are associated with obesity, type 2 diabetes, and CVD [144], representing an independent risk factor for CVD [145]. EVOO, particularly HT and OA, increases adiponectin level [146], supporting its cardiometabolic benefits.

*Plasminogen activator inhibitor-1 (PAI-1)*: PAI-1 is an independent risk factor for CVDs; it impairs fibrinolysis and contributes to thrombus formation [147]. Studies in humans [148] and in animals [149] showed that EVOO, particularly OLE, is a potent ligand of PAI-1 [78], indicating its potential benefits for thrombotic conditions.

*Proprotein convertase subtilisin/kexin type 9 (PCSK9)*: PCSK9 can irreversibly bind to LDL receptors, elevating its levels [150] and increasing the risk of atherosclerosis and cardiovascular events [151]. A trial study showed that the treatment with four capsules/day of EVOO, containing 2.5 mg HT each, for 1 month reduced PCSK9 levels [152], enhancing LDL clearance.

*Lipocalin-2* (LCN-2): LCN2 is a member of the lipocalin family, linked to inflammation and atherosclerosis. EVOO component, oleocanthal, has been shown to reduce LCN2 levels in human primary chondrocytes obtained from osteoarthritis patients [153].

### 4.5. Oxidative Stress Markers

*F2-Isoprostanes* (F2-IsoPs): F2-IsoPs, a class of bioactive compounds generated from arachidonic acid following oxidative stress, represent an independent risk factor for CHD [154]. A trial conducted among the older Australian population has demonstrated the reduction in F_2_-IsoPs subsequent to EVOO consumption [155]. In addition, a study in vitro has proven the inhibitory effect of HT on F2-IsoP synthesis following LDL oxidation [156], emphasizing its antioxidant capacity.

The effects of EVOO consumption on CVDs Biomarker are summarized in Supplementary Materials Table S5.

## 5. Heart-Healthy Composition of Extra Virgin Olive Oil: Fatty Acids, Bioactive Compounds, and Mechanisms of Action

EVOO is rich in MUFAs, particularly OA, known for improving the lipid profiles. EVOO also contains PUFAs, such as linoleic and alfa linolenic acid, along with saturated fatty acids, like palmitic acid, stearic acid, arachidic acid, and myristic acid, which contribute to its anti-inflammatory and heart-protective properties. Additionally, EVOO contains a wide array of bioactive polyphenols [157], enhancing its anti-inflammatory, antioxidant, and anti-atherosclerotic effects. The anti-atherosclerotic properties of EVOO have been demonstrated in studies conducted on apolipoprotein E-deficient mice. Claro et al. demonstrated that the phenolic compounds were able to restore endothelial function and to reduce lipid accumulation within the atherosclerotic plaque. However, the reductions in macrophage accumulation and inflammatory markers were independent of the phenolic content [158]. Further, atherosclerosis progression was delayed in mice who were fed a diet rich in EVOO obtained through centrifugation process compared to mice that received EVOO obtained though standard process. These results suggest that, beyond the presence of phenolic compounds, the preparation of OO is crucial in determining its anti-atherosclerotic properties [159]. Along with fatty acids and polyphenols, various small molecules, including branched-chain amino acids (BCAAs), aromatic AA, acylcarnitine, glutamine/glutamate ratio, gut flora-related metabolites, urea cycle metabolites, and polar lipids, also play a significant role in these protective effects. However, we will focus on the molecular mechanism of OA, as the main compound in EVOO, and of polyphenols that, despite their small presence in EVOO, are responsible for significant effects on cardiovascular health.

*EVOO fatty acid composition*: OA, the main MUFA in EVOO, constitutes 55–83% of its fat content. It exerts a plethora of beneficial effects on cardiovascular health, including reducing BP, improving lipid profile, enhancing endothelial function, and mitigating insulin resistance. Mechanistically, OA promotes the elimination of excess cholesterol from cells through the activation of ATP-binding cassette protein A1 (ABCA1) [160]. It also prevents LDL oxidation, inhibits vascular smooth muscle cells proliferation, and protects from atherosclerosis [161]. OA lowers BP by its activity on the adrenergic α2A/D-adrenoreceptor/G protein/adenylyl cyclase-cAMP/PKA signaling [162] through modulating the membrane lipids composition [163]. OA’s anti-inflammatory effects include inhibition of the NF-kB pathway [164], and reduction of IL-1, IL-6, TNF-α, and COX-2 levels [165]. The anti-inflammatory effect promoted by OA are associated with decreased oxidative stress [166,167], enhanced NO synthesis, and improved endothelial function. Lastly, OA improves insulin sensitivity through PI3K-AKT activation [164] and increases adiponectin levels [146]. Collectively, the effects mediated by OA contribute to lowering the risk of CVDs.

*EVOO polyphenols compounds*: The quantity of polyphenols in EVOO varies between 50 to 1000 mg/kg, depending on tree variety, agronomic conditions, production processes, harvesting method, and period, as well as oil extraction and distribution [168]. Further, polyphenols are sensitive to heat, light, and air [169], and they can lose up to 50% of their potency in 12 months if not stored properly. The polyphenols found in EVOO are classified in thirty-six different chemical classes, including secoiridoids, phenolic alcohols, phenolic acids, lignans, and flavones [170]. The health benefits of polyphenols are recognized by the European Food Safety Authority (EFSA) and have been stated in the EU health claim regulation, affirming that EVOO, containing at least 5 mg of HT and its derivatives (OLE complex and tyrosol) per 20 g, protects lipids from oxidative stress [171]. The main polyphenols in EVOO are derivatives of tyrosol (oleocanthal and ligstroside aglycone) and HT (oleacein and oleuropein aglycones); they can be either hydrolyzed in the gastrointestinal tract or can be directly absorbed by the intestine, depending on their chemical structure. However, the bioavailability in the plasma varies between 0 and 4.0 µmol/l after the oral consumption of 50 mg aglycone equivalents of a polyphenol [172]. This finding evidences the poor intestinal absorption and fast biotransformation that favors their urinary excretion. Following their absorption, polyphenols enter the bloodstream, targeting the whole body and modulating anti-inflammatory, antioxidant, vasodilatory, hypoglycemic, lipid-modulating, and gene-regulated pathways, collectively contributing to cardiovascular protection [173].

Polyphenols exert antioxidant and anti-inflammatory mechanisms. They counteract oxidative stress by directly scavenging ROS [174]; chelating metals [175]; and upregulating antioxidant enzymes such as catalase (CAT), thioredoxin reductase (TrxR), and heme oxygenase-1 (HO-1) [176]. The anti-inflammatory effects include the inhibition of NF-kB [177], which has been associated with a reduction in inflammatory factors, including interleukin-1, intercellular cell adhesion molecule-1, TNF-α, and MCP-1 [178]. Further, polyphenols have been demonstrated to suppress IL-6, microsomal PGE synthase-1 (mPGES-1) [179,180], and particularly, oleocanthal has been shown to inhibit COX-1 and COX-2 with a potency similar to that of ibuprofen [181].

Interestingly, polyphenols can also influence cardiovascular health indirectly by interacting with the gut microbiota. They function as prebiotics, enhancing beneficial bacteria like Lactobacillus and Bifidobacterium [182] while reducing harmful bacteria [183]. The microbiota, in turn, metabolizes polyphenols into bioactive form (mainly HT, oleuropein aglycone, and oleocanthal) that exert beneficial effects on health [184].

Emerging evidence highlights the endothelium as a significant target for EVOO polyphenols, which exert modulatory effects on its diverse functions. In particular, polyphenols reduce VEGF-induced angiogenic responses and NADPH oxidase, inhibiting Nox2, Nox4, MMP-2, and MMP-9 [185]. Moreover, HT and its derivates inhibit the endothelial adhesion molecules VCAM-1 and ICAM-1, conferring atheroprotection [186,187]. Polyphenols also promote NO bioavailability [45] through the activation of PI3KT/AKT pathway [188] and vasodilation trough the NO-cGMP pathway [189] and Ca^2+^-activated K^+^ channels [190]. In the study conducted by Gonzalez-Correa et al., it was shown that administration of HT and its derivate, HT-acetate increased NO production and decreased thromboxane synthesis in rats, mechanism through which contribute to inhibiting platelet aggregation [191]. Polyphenols may also exert vasorelaxation in an endothelium-independent manner through activation of L-type calcium channel and BK channels [192,193]. However, some polyphenols [194], such as quercetin [195], luteolin [196], and derivatives from oleuropein [197] have been shown to act through more than one of the aforementioned mechanisms [197]. Interestingly, 3,4-DHPEA-EA and 3,4-DHPEA-EDA have been shown to induce an endothelium-dependent mechanism of action at lower concentration towards an increase in NO, while exhibiting an endothelium-independent mechanism of action at higher concentration, mediated by the reduction of cytosolic calcium [197].

**Figure 2:**
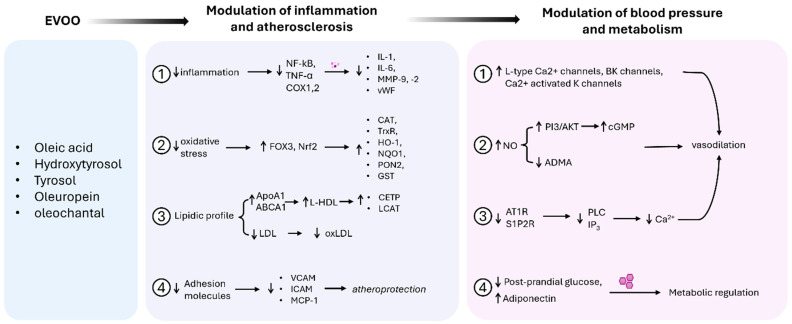
Mechanism of action of EVOO compounds. The diagram illustrates the beneficial effects of EVOO on cardiovascular health, highlighting its impact on inflammation, oxidative stress, lipid profile, and vascular function. Oleic acid, HT, and oleocanthal, contribute to reducing inflammatory markers, improving antioxidant defenses, and modulating blood pressure and glucose metabolism. This combination of effects supports cardiovascular protection and metabolic regulation. Nuclear Factor kappa B (NF-κB), Tumor Necrosis Factor-alpha (TNF-α), Cyclooxygenase-1 and -2 (COX1,2), Interleukin-1 (IL-1), Interleukin-6 (IL-6), Matrix Metalloproteinase 9 and 2 (MMP-9, -2), von Willebrand Factor (vWF). Forkhead Box O3 (FOXO3), Nuclear factor erythroid 2-related factor 2 (Nrf2), Catalase (CAT), Thioredoxin Reductase (TrxR), Heme Oxygenase-1 (HO-1), NAD(P)H Quinone Dehydrogenase 1 (NQO1), Paraoxonase 2 (PON2), Glutathione S-transferase (GST), Apolipoprotein A1 (ApoA1), ATP-binding cassette transporter A1 (ABCA1), Cholesteryl Ester Transfer Protein (CETP), Lecithin–Cholesterol Acyltransferase (LCAT), low-density lipoprotein (LDL), oxidized low-density lipoprotein (oxLDL), high-density lipoprotein (HDL), Vascular Cell Adhesion Molecule 1 (VCAM-1), Intercellular Adhesion Molecule 1 (ICAM-1), Monocyte Chemoattractant Protein-1 (MCP-1), L-type Calcium Channels (L-type Ca^2+^ channels), Big Potassium Channels (BK channels), Calcium-activated Potassium Channels (Ca^2+^ activated K channels), Phosphoinositide 3-Kinase/AKT Pathway (PI3K/AKT), Cyclic Guanosine Monophosphate (cGMP), Asymmetric Dimethylarginine (ADMA), Angiotensin II Receptor Type 1 (AT1R), Sphingosine-1-Phosphate Receptor 2 (S1P2R), Phospholipase C (PLC), and Inositol 1,4,5-Trisphosphate (IP_3_). ↑ = increase; ↓ = decrease.

EVOO’s cardiovascular benefits mediated by its compounds are summarized in Figure 2.

## 6. Future Directions and Implications

The antioxidant, anti-hypertensive and anti-inflammatory as well as anti-atherogenic and anti-thrombotic effects define EVOO beneficial cardiovascular properties. OA and polyphenolic compounds such as HT and OLE are responsible for these effects. However, more detailed investigations are needed to identify the contribution of the specific bioactive compounds, and their effects on gene expression, cellular signaling pathway and enzyme activities on cardiovascular health. Also, a comprehensive understanding of how genetic, environmental and lifestyle factors influence the efficacy of EVOO should be further studied.

Technological advancements have enormous potential for improving the quality and efficacy of EVOO; in this regard, genetic engineering may be a novel approach to develop olive cultivars with higher concentrations of polyphenols. Also, the possibility of enhancing extraction techniques ensuring sustainable production practices, guaranteeing the safety and authenticity of EVOO products, should be further investigated. Lastly, the production of nutraceutical EVOO-based that concentrate specific bioactive compounds and enhance the bioavailability employing advanced delivery system (e.g., liposomes or nanoparticles) may be another opportunity. Furthermore, raising awareness about the proper use and selection of EVOO, along with providing guidelines for its integration into a heart-healthy diet, could not only maximize cardiovascular benefits but also help in reducing healthcare system costs. The number demonstrated that increasing the adherence to a Mediterranean dietary pattern by 20% would produce annual savings in cardiovascular-related cost by USD 8.2 billion in the US [198].

## 7. Conclusions

CVDs are a chronic, lifestyle-related illness since nutrition, physical activity, and stress levels participate in its development. The constellations of pathophysiological contributors to CVDs include hypertension, dyslipidemia, inflammation, oxidative stress, and hyperglycemia. This multifactorial nature of CVDs complicates the traditional treatment strategies. Indeed, common medications target single pathways, not fully addressing the complex pathophysiology behind CVDs development. Although the cardioprotective effects of EVOO are consistently mixed with evidence provided by other components of the dietary pattern, such as the MedDiet, the difficulty of separating the specific effects of EVOO is acknowledged. Nevertheless, a large body of evidence conducted on individual bioactive compounds found in EVOO has consistently demonstrated their efficacy in modulating the pathways implied in CVDs development. These findings strongly support the role of EVOO in promoting cardiovascular health. In conclusion, a diet high in EVOO is compatible with healthy arteries and a lower risk of cardiovascular events. The evidence proves that EVOO is a healthy dietary fat, and consuming high polyphenols EVOO may be even more beneficial in maintaining cardiovascular homeostasis.

## Data Availability

Data sharing is not applicable.

## Supplementary Materials

The following supporting information can be downloaded at: https://www.mdpi.com/article/10.3390/biom15020284/s1, Table S1: Summarize the key studies discussed in section “MedDiet benefits on cardiovascular problems”; Table S2: Summarize the key studies discussed in section “EVOO and cardiovascular health: Evidence and Benefits”; Table S3: Summarize the key studies discussed in section “EVOO impacts on Arterial Health and Hemostasis”; Table S4: Summarize the key studies discussed in section “EVOO impact on hemostasis”; Table S5: Summarize the Effects of EVOO consumption on CVDs Biomarker.

## List of Abbreviations

BP	blood pressure
CHD	coronary heart disease
DBP	diastolic blood pressure
EVOO	extra virgin olive oil
FMD	flow-mediated dilation
HDL	high-density lipoprotein
HT	hydroxytyrosol
IMT	intima-media thickness
LDL	low-density lipoprotein
MedDiet	Mediterranean diet
MUFA	monounsaturated fatty acid
NO	nitric oxide
OA	oleic acid
Ole	oleuropein
OO	olive oil
PUFAs	polyunsaturated fatty acids
SBP	systolic blood pressure
TXA2	thromboxane A2
TXB2	thromboxane B2
VOO	virgin olive oil
