# Supplementary material for: Exploring the Cardiovascular Benefits of Extra Virgin Olive Oil: Insights into Mechanisms and Therapeutic Potential
Source: Biomolecules. 2025 Feb 14;15(2):284. doi: 10.3390/biom15020284 (PMC11852600; doi:10.3390/biom15020284)
Supplement: Supplementary file 1 [file biomolecules-15-00284-s001.zip › biomolecules-3424033-supplementary.pdf]

Supplement materials

Table S1. Summarize the key studies discussed in section " MedDiet benefits on cardiovascular problems" . The arrows ↑ and ↓ indicate increase and reduction respectively.

Referen.	Study Type	Campion and health condition at baseline	Intervention Type	Follow-Up	Outcome Measured	Major Results	Major Scientific Limitations
[12]	Prospective Cohort Study	22,043 adult men and women healthy	Assessment of adherence to a MedDiet using a 10-point scale	3.6 years	Mortality from all causes.	- MedDiet is associated with a reduction in total mortality<br>• HR for death per two-point increase in the Mediterranean-diet score: 0.75 (95% CI: 0.64–0.87).<br>- Inverse associations observed for cause-specific mortality:<br>• Coronary heart disease: Adjusted HR: 0.67 (95% CI: 0.47–0.94).	potential confounding by lifestyle factors.
[16]	Prospective Cohort Study	11,579 men, 40–59 years healthy	Observational study on diet and lifestyle	15 years	CV mortality rate	MedDiet associated with lower cardiovascular mortality (HR 0.60, CI 0.50-0.70)	Limited to men, excluding women from analysis.
[15]	RCT	7,447 participants; men (55–80 years) and women (60–80 years)	MedDiet +olive oil (1l/weeks) or nuts (30g).	4.8 years	CV events; CV mortality	- CVD events (composite of myocardial infarction, stroke, or CVD death; 288 events over 4.8 years):<br>• Hazard ratio (HR): 0.70 (95% CI: 0.53–0.91) for MeDiet+EVOO vs. control.<br>- Incident diabetes (273 cases among 3541 non-diabetic participants):<br>• HR: 0.60 (95% CI: 0.43–0.85) for MeDiet+EVOO vs. control.<br>- Improvements in CVD risk factors	Generalizability limited to high-risk populations.
[18]	RCT	605 participants; post-myocardial infarction patients.	MedDiet +alpha-linolenic acid.	4 years	Composite outcomes (COs) studied:<br>CO 1: Cardiac death and nonfatal myocardial infarction.<br><br>CO 2: CO 1 + major secondary endpoints	• CO1: 14 events vs. 44 in the prudent Western-type diet group (P=0.0001).<br>• CO 2: 27 events vs. 90 (P=0.0001).<br>• CO 3: 95 events vs. 180 (P=0.0002).	Small sample size, self-reported adherence to MedDiet

					CO 3: CO 2 + minor events requiring hospital admission.		
[19]	RCT	605 participants < 70 years old	MedDiet +alpha-linolenic acid.	5 years	coronary events; coronary heart disease mortality	No difference in Serum lipids, blood pressure, and body mass index.<br>• Cardiac deaths: 16 in the control vs. 3 in the experimental group.<br>• Non-fatal myocardial infarctions: 17 in the control vs. 5 in the experimental group.<br><br>↓Overall mortality:<br>• 20 deaths in control vs. 8 in experimental group.	use of a margarine with comparable composition to olive oil fatty acid
[20]	secondary, randomized, prospective prevention trial	574 participants primarly men, (mean age 53.5). Established myocardial infarction	MedDiet+alpha-linolenic acid.	5 years	incidence of coronary artery disease	• ↓70-80% of primary clinical endpoints and secondary CV endopints<br>• ↓70% in total death	use of a margarine with comparable composition to olive oil fatty acid
[21]	Meta-Analysis	12 studies included; General population and high-risk groups	Adherence to the MedDiet.	Varies by study (3–18 years)	CV events; CV mortality;	↓9% in overall mortality and CVDs mortality	Heterogeneity among studies included in the analysis
[22]	Prospective Cohort Study	25.994 post menopausal women, (mean 54,7 years) No pre-existing CVD	Assessment of adherence to a MedDiet using a 10-point scale	12 years.	• CV diseases incidence<br>• biomarker changes	↓25% CVD risk with higher adherence to MedDiet	Limited to postmenopausal women

Table S2. Summarize the key studies discussed in section "EVOO and cardiovascular health: Evidence and Benefits". The arrows ↑ and ↓ indicate increase and reduction respectively.

Referen.	Study Type	Campion and health condition at baseline	Intervention Type	Follow-Up	Outcome Measured	Major Results	Major Scientific Limitations
[28]	RCT	3,541 participants; men (55–80 years) and women (60–80 years)<br>High cardiovascular risk without diabetes	MedDiet +EVOO (1l/weeks) or nuts (30g).	4.1 years	Incidence of type 2 diabetes	- Diabetes incidence:<br>• MedDiet +EVOO: 10.1% (95% CI: 5.1–15.1).<br>• MedDiet + nuts: 11.0% (95% CI: 5.9–16.1).<br>• Control group: 17.9% (95% CI: 11.4–24.4)	Sekf-reported adherence to the MedDiet; Partecipants were not blinded
[29]	Prospective Cohort Study	22,295 participants; adults from eight European countries.<br>No pre-exhisting diabetes	Assessment of adherence to a MedDiet using a 18-point scale	12 years	Incidence of type 2 diabetes.	Type 2 diabetes risk reduction with adherence to the Mediterranean diet (MDP):<br>• Medium adherence (rMED 7–10 points): Hazard ratio (HR): 0.93 (95% CI: 0.86–1.01).<br>• High adherence (rMED 11–18 points): HR: 0.88 (95% CI: 0.79–0.97).<br>• Reference group: Low adherence to MDP (rMED 0–6 points).<br>• P for trend = 0.013.	adherence categories might oversimplify the continuum of dietary practices.
**[49]**	RCT	322 individuals (mean ages 52 years); moderately obese	MedDiet+OO (30-45g) vs Low carb vs Low fat	2 years	• weight loss<br>• glycemic control<br>• lipid profile	- Weight loss:<br>• (MedDiet): 4.4 kg.<br>• Low-carb diet: 4.7 kg.<br>• Low-fat diet: 2.9 kg.<br>- ↓Waist circumference MedDiet: 3.5 ± 5.1 cm.<br>↓SBP: - 5.5 ± 14.3 mmHg in the MedDiet group.<br>↓DBP: - 2.2 ± 9.5 mmHg in the MedDiet group.	Self-reported adherence; The low-carbohydrate group often experiences rapid initial weight loss due to water loss associated with glycogen depletion.

						- Inflammatory markers:<br>• ↓21%High-sensitivity C-reactive protein: in the MedDiet group.<br>• ↑Leptin in the MedDiet group.<br>- Glycemic and insulin measures:<br>• ↓Fasting plasma glucose: by 32.8 mg/dL.<br>• ↓HOMA-IR in the MedDiet group after 24 months.<br>• ↓Glycated hemoglobin by 0.5 ± 1.1% after 24 months in the MedDiet group.<br>- Liver enzyme levels:<br>• ↓Alanine aminotransferase (ALT) by 3.4 ± 11.0 in the MedDiet group over 24 months.<br>- Lipid profile changes (24 months):<br>• ↓LDL cholesterol by 5.6 mg/dL.<br>• ↑HDL cholesterol by 6.3 mg/dL.<br>• ↓Triglycerides by 21.8 mg/dL.<br>• ↓Total cholesterol to HDL ratio by 0.9.	
[32]	RCT	3541 patients aged 55 to 80 years	MedDiet+EVOO vs MedDiet+nuts vs low fat diet	4 years	Incidence of type 2 diabetes.	New-Onset Diabetes Cases (per 1000 person-years):<br>• EVOO: 1.6%<br>• Nuts 1.87%<br>• Control 2.36%<br>Multivariate-Adjusted Hazard Ratios:<br>• EVOO group: HR 0.60 (95% CI, 0.43–0.85)<br>• Nuts group: HR 0.82 (95% CI, 0.61–1.10)	Randomization was not stratified by diabetes status. Withdrawals were greater in the control group.

[33]	RCT	10 non-insulin-dependent diabetic patients aged 52 +/- 8 year	high-MUFA/low-CHO diet vs low-MUFA/high-CHO diet	15 days	• insuline sensitivity<br>• lipid profile	High MUFA vs low-CHO<br>• ↓Postprandial Glucose 8.76 ± 2.12 vs. 10.08 ± 2.76 mmol/L; *P* < .05.<br>• ↓Plasma Insulin 195.0 ± 86.4 vs. 224.4 ± 75.6 pmol/L; *P* < .02.<br>• ↓Fasting Triglycerides 1.16 ± 0.59 vs. 1.37 ± 0.59 mmol/L; *P* < .01.<br>• ↑Insulin-Mediated Glucose Disposal 5.8 ± 2.1 vs. 4.6 ± 1.8 mg/kg/min; *P* = .02	Short intervention period
[34]	RCT	12 women with NIDDM.	High-MUFA vs high-carbohydrate. (MUFA from OO and avocado)	8 wks	• glicemic control<br>• lipid profile	• Both diets no significant change in HDL cholesterol.<br>• ↓ plasma triglycerides in HMUFA diet (20% vs. 7% for high-CHO diet).<br>• Glycemic control was similar between both diets.	results specific to NIDDM.
[35]	RCT	42 patients (33 men and 9 women) 35-78 aged NIDDM	High-MUFA vs high-carbohydrate. (OO was used as the main source of fat)	12-20 weeks	• glicemic control<br>• lipid profile	• The high-carbohydrate diet increased fasting plasma triglycerides and VLDL cholesterol by 24% (P<.0001) and 23% (P=.0001), respectively.<br>• It also raised day-long plasma triglycerides, glucose, and insulin by 10% (P=.03), 12% (P<.0001), and 9% (P=.02), respectively.<br>• Plasma total cholesterol, LDL, and HDL cholesterol levels remained unchanged.<br>• The effects on plasma glucose, insulin, and triglycerides persisted for 14 weeks.	results specific to NIDDM.
[36]	RCT	18 patients with either rmild or	high-carbohydrate/low-fiber	30 days	Glycemic control (HbA1c,	• The high-carbohydrate diet significantly	Small sample size;

		severe glucose intolerance	vs a low-carbohydrate/low-fiber diet (40% energy from carbohydrate and 40% from fat) MUFA mainly from OO		fasting glucose) Insulin sensitivity	increased postprandial blood glucose in patients on glibenclamide (13.6 ± 1.4 vs. 11.0 ± 1.8 mmol/l, P<0.002), but not in the diet-only group (9.7 ± 0.7 vs. 8.9 ± 0.6 mmol/l).<br>• Postprandial insulin was higher in the diet-only group (248 ± 32 vs. 192 ± 28 pmol/l, P<0.01), but no significant differences were seen in the glibenclamide group (226 ± 19 vs. 202 ± 24 pmol/l).<br>• The high-carbohydrate diet increased fasting plasma triglycerides in both groups (1.36 ± 0.2 vs. 1.12 ± 0.2 mmol/l, P<0.05 and 1.4 ± 0.3 vs. 1.1 ± 0.1 mmol/l, P<0.05).<br>• No differences were observed in fasting plasma cholesterol or HDL levels.	Short intervention period
[37]	RCT	25 healthy subject	Study 1: MedDiet vs MedDiet+EVOO 10 g<br><br>Study 2: MedDiet+ EVOO (10 g) vs MedDiet+ corn oil (10 g)		Post-prandial blood glucose LDL cholesterol	Study 1:<br>• ↓blood glucose after 2 h with EVOO (P<0.001),<br>• ↓DPP-4 protein (P<0.001) and activity (P<0.001)<br>• ↓ LDL-C (P<0.001), and ox-LDL (P<0.001),<br>• ↑insuline (P<0.05), GLP-1 (P<0.001), and GIP (P<0.05)<br>Study 2:<br>• improved glycemic and lipid profiles compared to corn oil, with a smaller increase in glucose (P<0.05), DPP-4 protein (P<0.001) and activity (P<0.05), and a higher	Small sample size

						increase in insulin (P<0.001) and GLP-1 (P<0.001).<br>• increase in LDL-C (P<0.05) and ox-LDL (P<0.001) compared to corn oil.	
[38]	RCT	33 hypercholesterolemic individuals	1. VOO containing 80 ppm of phenolic compounds<br>2. FVOO: VOO + 500 ppm of phenolic compounds<br>3. FVOOT: VOO +500ppm of phenols (VOO+thyme 1:1)<br><br>Consumption: 25ml/daily	5 wks	Lipoprotein particle profiles<br>Atherogenic ratios	• No changes in glucose, triglycerides, total cholesterol, or HDL-C.<br>• ↓ LDL-C after FVOO<br>• No effect on blood pressure.<br>• ↓LDL-P, IDL-P, and ApoB-100 after FVOO.<br>• ↑L-HDL<br>• ↓s-HDL<br>• ↓VLDL after FVOO.<br>• ↓ LDL-P/HDL-P ratio, HDL-C/HDL-P ratio, and s-HDL/L-HDL ratio after FVOO.	Small sample size, Specificity of population
[42]	RCT	23 patients<br>10 male and 13 female; age range, 25-70 years hypertensive	MUFA (EVOO) vs PUFA (sunflower oil)	12 months	Blood pressure levels<br>Antihypertensive medication dosage	MUFA vs PUFA<br>• ↓Resting blood pressure (P = .05 for SBP; P = .01 for DBP).<br>• BP responses during sympathetic stimulation were similar for both diets.<br>• ↓Daily drug dosage (-48% vs -4%, P<.005).<br>• All patients on the PUFA diet required antihypertensive treatment, while 8 patients on the MUFA diet needed no drug therapy.	Small sample size;<br>Both sex-included introducing potential bias
[43]	RCT	162 patients with stage-1 hypertension	Olive Leaf Extract 500 mg, twice daily vs Captopril 12.5 mg, twice daily	12 weeks	Blood puressure	• ↓ SBP and DBP after 8 weeks<br>• ↓SBP was -11.5±8.5 mmHg in the Olive group and -	Small sample size

						13.7±7.6 mmHg in the Captopril group;<br>• ↓ DBP was -4.8±5.5 mmHg and -6.4±5.2 mmHg, respectively<br>• ↓triglyceride levels in the Olive group, but not in the Captopril group.	
[44]	double-blind, randomised, controlled, crossover trial	60 partecipants (24-72 years old) males; pre-hypertensive	OLE (136 mg oleuropein; 6 mg hydroxytyrosol) vs polyphenol-free	6 weeks	Blood pressure; Inflammatory markers	OLE:<br>• ↓Daytime SBP by 3.95 mmHg (P = 0.027),<br>• ↓ 24-h SBP by 3.33 mmHg (P = 0.045),<br>• ↓daytime DBP by 3.00 mmHg (P = 0.025),<br>• ↓24-h DBP by 2.42 mmHg (P = 0.039)<br>• ↓plasma total cholesterol (-0.32 mmol/L, P = 0.002),<br>• ↓LDL cholesterol (-0.19 mmol/L, P = 0.017),<br>• ↓triglycerides (-0.18 mmol/L, P = 0.008),<br>• ↓interleukin-8 (-0.63 pg/ml, P = 0.026).<br>• No effects on other markers of inflammation, vascular function, or glucose metabolism.	Smal sample size, not stratified by age
[45]	double-blind, randomized, crossover	24 young women (24–27 years) high-normal BP or stage 1 essential hypertension	EVOO polyphenol-rich (564 mg/kg polyphenols), vs polyphenol-free consumption: 60 ml daily ~30 mg/day polyph	2 months	Blood pressure; Inflammatory markers	EVOO polyphenol-rich:<br>• ↓ SBP by 7.91 mmHg (P < 0.01) and DBP by 6.65 mmHg.<br>• ↓ serum ADMA (-0.09 μmol/l, P < 0.01),<br>• ↓ox-LDL (-28.2 μg/l, P < 0.01),<br>• ↓CRP (-1.9 mg/l, P < 0.001).<br>• ↑plasma nitrites/nitrates (+4.7 μmol/l, P < 0.001)	Small sample size, limited to women

						• ↑hyperemic area after ischemia (+345 perfusion units/sec, P < 0.001).	
[46]	randomized, single-blinded and placebo-controlled trial	41 participants (aged ≥ 65) overweight or obese	control (mixture of corn, soybean oil and butter) vs EVOO	3 motnhs	Blood pressure Immunitary response	• ↓SBP (P < 0.05),<br>• ↑ plasma HDL-C (P = 0.06),<br>• ↑anti-CD3/anti-CD28-stimulated T cell proliferation (P < 0.05) compared to the CON group.<br>• No differences were found in T cell phenotype, cytokine production, or DTH response between the groups.	Small sample size, participants were given oil/spread for *ad libitum* use in their cooking at home
[48]		7447 asymptomatic men (aged 55-80 years) and women (aged 60-80 years) who had type 2 diabetes or three or more cardiovascular risk factors	MedDiet+EVOO (1l/week) or MedDiet+nuts (30g)	5 years	Antrophometric measurment	MedDiet+EVOO vs nuts<br>• ↓ body weight of -0.410 kg (P = 0.056), while the nut group showed no significant change (-0.016 kg, P = 0.942), compared to the control group.<br>• MedDiet+EVOO ↓ waist circumference of -0.466 cm (P = 0.154), while the nut group showed a significant reduction of -0.923 cm (P = 0.008), compared to the control group.	Focused on secondary outcomes; primary cardiovascular results may influence interpretations.
[54]	RCT	47 healthy European male	OO polyphenol-poor (2.7 mg/kg) vs OO polyphenol-rich (366 mg/kg) consumption: 25 mL/d	3 weeks	Lipidic profile Cholesterol efflux	polyphenol-rich vs polyphenol-poor<br>• ↑HDL cholesterol efflux capacity after the polyphenol-rich intervention (+3.05%) compared to the polyphenol-poor one (-2.34%, P = 0.042).<br>• ↑ levels of large HDL (HDL2), compared to the polyphenol-poor intervention.	Small sample size;

						• ↓ Small HDL (HDL3) levels,<br>• HDL core became triglyceride-poor,<br>• ↑ HDL fluidity	
[53]	multi-center random-ized, cross-over, clini-cal trial	200 male Euro-pean subjects (mean age: 33.1) healthy	EVOO with high (HPC, 366 mg/Kg), me-dium (MPC, 164 mg/Kg), and low (LPC, 2.7 mg/Kg) phe-nolic content	3 wks	Lipidic profile; Lipoprotein oxidation	• ↑oleic acid in LDL by 1.9% ($p < 0.001$),<br>• ↓linoleic acid by 1.1% ($p < 0.002$)<br>• ↓arachidonic acid by 0.5% ($p < 0.001$).<br>• ↑MUFA/PUFA and oleic/linoleic acid ratios in LDL<br>• inverse rela-tionship between oleic/linoleic acid ratio and biomarkers of oxi-dative stress.	
[52]	Random-ized, crosso-ver, con-trolled trial.	200 male Euro-pean subjects (mean age: 33.1 healthy	EVOO with high (HPC, 366 mg/Kg), me-dium (MPC, 164 mg/Kg), and low (LPC, 2.7 mg/Kg) phe-nolic content	3 weeks	Lipoprotein oxidation	• ↑ HDL levels linearly with increas-ing phenolic content<br>• ↓ total choles-terol/HDL cholesterol ratio linearly with higher phenolic con-tent<br>• ↓Triglyceride levels by 0.05 mmol/L across all olive oils.<br>• ↓Oxidative stress markers linearly with increasing phe-nolic content.<br>• Oxidized LDL levels showed a greater reduction with higher polyphenol content:	Dietary in-take self-re-ported

Table S3. Summarize the key studies discussed in section "EVOO impacts on Arterial Health and Hemostasis". The arrows ↑ and ↓ indicate increase and reduction respectively.

Referenc.	Study Type	Campion and health condi-tion at baseline	Intervention Type	Follow-Up	Outcome Measured	Major Results	Major Scien-tific Limita-tions

[57]	RCT with a cross-over design	10 subjects with type 1 diabetes and 6 healthy subjects age ≥ 18 years	HGI + EVOO (35g)<br>HGI +butter (40g)	1-5h post meal	FMD<br>Glucose<br>Lipid profile	EVOO vs Butter:<br>• ↑ FMD (P = 0.007).<br>• EVOO improved vascular function, whereas butter had detrimental effects.	Small sample size; markers of NO bioavailability in the post-prandial state not included; short outcome
[58]	systematic review and meta-analysis	30 studies enrolling 3106 participants	OO consumtpion (1mg and 50 mg)		Inflammatory markers; FMD	OO vs control<br>• ↓ C-reactive protein (-0.64 mg/L, P < 0.0001, n = 15 trials)<br>• ↓ interleukin-6 (-0.29, P < 0.04, n = 7 trials)<br>• ↑ FMD (+0.76%, P < 0.002, n = 8 trials).	Heterogeneous study design
[59]	RCT	805 CHD patients	MedDiet (MUFAs) vs low-fat diet (MUFA from olive oil consumption >4 tablespoons/day; 10–15 g/tablespoon)	1 year	FMD,	MUFAs vs low-fat diet:<br>• ↑ FMD (3.83% vs. 1.16%, P = 0.011), ↑endothelial progenitor cells (EPCs) (+1.64%, P = 0.028)<br>• ↓ endothelial microparticles (EMPs) (-755 EMPs/μl, P = 0.015)<br>• ↓ ROS production (-11.1, P = 0.010), ↓cellular apoptosis (-20.2, P = 0.013),<br>• ↓ senescence (-18.0, P = 0.031),<br>• ↑ cellular proliferation (+11.3, P = 0.011)<br>• ↑ angiogenesis (+549 master segments length, P = 0.022).	secondary endpoint of the CORDIOPREV study;
[63]	RCT	1,002 participants (aged 55-75 years) coronary heart disease (CHD)	Med-Diet+EVOO vs low-fat diet	5-7 years	Changes in carotid intima-media thickness (IMT)	• decreased IMT-CC at 5 years (−0.027±0.008 mm; P<0.001), maintained at 7 years (−0.031±0.008 mm; P<0.001),	secondary end-point of the CARDIOPREV study

						• slower progression of coronary artery disease and reduction in coronary artery calcium scores.<br>• improvements in lipid profiles and inflammatory markers	
[64]	Observational Study	199 participants(mean age 67.3 years) high cardiovascular risk	olive oil consumption ranged from 6 to 74 g/day, categorized in energy-adjusted quin- tiles to explore its association with the carotid IMT.	4 years	measurements of carotid intima-media thickness (IMT), blood pressure, lipid profile, glucose levels	inverse association between olive oil consumption and carotid intima-media thickness	Secondary endpoint of the PREDIMED study
[65]	RCT	175 partecipants High cardiovascular risk	MedDiet +extra-virgin olive oil (1l/week) MedDiet +nuts (30g) vs low fat diet	2.4 years	Changes in carotid intima-media thickness (IMT)	no changes in ICA-IMT or plaque after the MedDiet+EVOO	Relatively small number; inclusion of confounding variables in the multivariate analyse;

Table S4. Summarize the key studies discussed in section "EVOO impact on hemostasis"**.** The arrows ↑ and ↓ indicate increase and reduction respectively.

Ref	Study Type	Campion and health condition at baseline	Intervention Type	Follow-Up	Outcome Measured	Major Results	Major Scientific Limitations
[67]	clinical trial	26 partecipants (aged 39-65) high cardiovascular risk	MedDiet+olive oil vs corn oil	8 wks	Changes in plasma lipid levels; Changes in platelet aggregation	• HDL were unchanged or raised by olive.<br>• Plasma apolipoprotein B levels were equally reduced by both diets;<br>• ↑apolipoprotein AI and the apo AI:B ratio with olive oil.<br>• ↓Plasma-glucose levels with olive oil.	Small sample size

						• ↓sensitivity to to collagen with olive.	
[68]	Pilot Study	7 partecipants healthy subjects	21g olive oil daily	8 wks	Hemostatic factors	• no significant changes in platelet count, platelet volume, concentrations of plasma fibrinogen, serum cholesterol, LDL and HDL<br>• Platelet membrane oleic acid content increased significantly ($P<0.01$),<br>• arachidonic acid content decreased significantly ($P<0.05$).	Small sample size
[71]	RCT	22 partecipants mildly dyslipidemic subjects	40 mL/day of either extra-virgin (phenol rich), or refined phenol poor (ROO)	4 wks	cardioprotective potential and vascular function.	• No effects on plasma lipid/lipoprotein profile<br>• ↓ serum TXB2 production and 8-iso-PGF2a<br>• ↑plasma antioxidant capacity	Small sample size;
[73]	RCT	14 partecipants (men) healthy subjects	diets enriched in refined olive oil (ROO); high-palmitic sunflower oil (HPSO), butter, or a mixture of vegetable and fish oils (VEFO)	10 wks	effects on heomostatic system	• oleic to palmitic acid ratio in the diet was associated with reduced thrombogenic factors<br>• a higher oleic acid diet promoted fibrinolytic activity	Small sample size, Limited to men
[74]	RCT	38 healthy volunteers (20 women, 18 men; mean age 27)	high LA diet (11.5 en%) or a high OA diet (18.0 en%)	8 wks	effects on coagulation and fibrinolytic factors	• No differences between the OA and LA diets were found in the plasma levels of fibrinogen, plasminogen activator inhibitor, antithrombin III, von Willebrand factor antigen or D-dimers.<br>• Factor FVII coagulant activity was significantly lower after the OA diet.	Small sample size
[75]	RCT	44 subjects (mean age 49) hypertensive subjects	extra-virgin olive oil (MUFA) or soy oil (PUFA) diet	3 months	effects on heomostatic system	• no difference in the levels of total cholesterol, triglycerides and BMI.<br>• ↓ DBP in both groups with respect to baseline values.<br>• ↓PAI-1 and F1+2 in the MUFA diet	Small sample size
[76]	RCT	25 partecipants (men) healthy subjects	low fat NCEP-I-diet MUFA-diet	84 days	effects on endothelial function	• ↓vWF, PAI-1 and TFPI plasma levels	Small sample size

			SAT rich-diet (SAT-diet)		• ↑ lag time of conjugated diene formation after the MUFA-diet.<br>• positive correlation between the decreases in TFPI and vWF and the changes in total cholesterol, LDL-C, apo B plasma level<br>• PAI-1 plasma levels were positively correlated with total cholesterol, LDL-C and triglycerides and negatively correlated with HDL-C.
[79]	Experimental (In vitro)	Human ER-/PR- breast cancer cell lines	oleuropein	Proliferation rate of breast cancer cells. PAI-1 expression levels. Changes in molecular signaling pathways related to cell proliferation and apoptosis.	EVOO or oleuropein treatment alone act as a natural PAI-1 inhibitor destabilizing downstream caspase activation and cell growth inhibition

Table S5. Summarize the Effects of EVOO consumption on CVDs Biomarker.

Biomarker	Category	Evoo effects
Aspartate aminotransferase AST/ALT ratio	Cellular Damage Markers	Animals: Reduction [93]<br><br>Human: reduction [89] [90,91]
Lactate dehydrogenase (LDH)	Cellular Damage Markers	Animals: Reduction [93]
Creatine kinase-MB (CK-MB)	Myocardial Damage Markers	Animals: reduction [94]
(NT-proBNP)	Cardiac Stress Markers	Human: reduction [108]
Cardiac Troponin	Myocardial Damage Markers	Animals: Inverse association [102]
C reactive protein (CRP) and IL-6	Inflammatory Markers	Human: reduction [58]
Tumour necrosis factor (TNF-α)	Inflammatory Markers	Human: reduction [7]<br><br>Animals: reduction [115,116]
Soluble CD40 Ligand	Inflammatory Markers	Human plateles: reduction [121]
F2 Isoprostanes	Oxidative Stress Markers	Human plasma samples: reduction [156]
Matrix metalloproteinases (MMP)	Tissue Remodeling Markers	Human vascular endothelial cells: inhibition [125]
Galectin-3	Fibrosis Markers	Bladder cacer cell: reduction [133]
Trimethylamine n-oxide (TMAO)	Metabolic Markers	Humans: reduction [141] [143,144]
MMP9/Neutrophil gelatinase-associated lipocalin (NGAL)	Oxidative Stress Markers	Atherosclerotic plaque: decrease [130]
Adiponectin	Metabolic Markers	Cells: prevent the reduction [147]

PAI-1 (Plasminogen activator inhibitor 1)	Coagulation Markers	Human: reduction [149]<br>Animals: reduction [78]<br>Cells: inhibition [79]
Proprotein convertase subtilisin/kexin type 9 (PCSK9)	Metabolic Markers	Humans: reduction [152]
Lipocalin 2	Inflammatory Markers	Cells: reduction [153]
